# Perinatal Exposure of Mice to the Pesticide DDT Impairs Energy Expenditure and Metabolism in Adult Female Offspring

**DOI:** 10.1371/journal.pone.0103337

**Published:** 2014-07-30

**Authors:** Michele La Merrill, Emma Karey, Erin Moshier, Claudia Lindtner, Michael R. La Frano, John W. Newman, Christoph Buettner

**Affiliations:** 1 Department of Environmental Toxicology, University of California Davis, Davis, California, United States of America; 2 Department of Preventive Medicine, Mount Sinai School of Medicine, New York, New York, United States of America; 3 Metabolism Institute, Mount Sinai School of Medicine, New York, New York, United States of America; 4 Division of Endocrinology, Diabetes and Bone Disease, Mount Sinai School of Medicine, New York, New York, United States of America; 5 West Coast Metabolomic Center, University of California Davis, Davis, California, United States of America; 6 Department of Nutrition, University of California Davis, Davis, California, United States of America; 7 Obesity and Metabolism Research Unit, USDA-ARS-Western Human Nutrition Research Center, Davis, California, United States of America; Univ Mississippi Medical Center, United States of America

## Abstract

Dichlorodiphenyltrichloroethane (DDT) has been used extensively to control malaria, typhus, body lice and bubonic plague worldwide, until countries began restricting its use in the 1970s. Its use in malaria control continues in some countries according to recommendation by the World Health Organization. Individuals exposed to elevated levels of DDT and its metabolite dichlorodiphenyldichloroethylene (DDE) have an increased prevalence of diabetes and insulin resistance. Here we hypothesize that perinatal exposure to DDT disrupts metabolic programming leading to impaired metabolism in adult offspring. To test this, we administered DDT to C57BL/6J mice from gestational day 11.5 to postnatal day 5 and studied their metabolic phenotype at several ages up to nine months. Perinatal DDT exposure reduced core body temperature, impaired cold tolerance, decreased energy expenditure, and produced a transient early-life increase in body fat in female offspring. When challenged with a high fat diet for 12 weeks in adulthood, female offspring perinatally exposed to DDT developed glucose intolerance, hyperinsulinemia, dyslipidemia, and altered bile acid metabolism. Perinatal DDT exposure combined with high fat feeding in adulthood further impaired thermogenesis as evidenced by reductions in core temperature and in the expression of numerous RNA that promote thermogenesis and substrate utilization in the brown adipose tissue of adult female mice. These observations suggest that perinatal DDT exposure impairs thermogenesis and the metabolism of carbohydrates and lipids which may increase susceptibility to the metabolic syndrome in adult female offspring.

## Introduction

The prevalence of type 2 diabetes (T2D) is rising worldwide [1], creating a tremendous burden for health care systems in developed and developing nations. The estimated cost associated with T2D was $245 billion last year in the United States alone [2]. This rise in the incidence of T2D is largely attributed to increasing obesity rates [3]. Obesity and T2D are characterized by insulin resistance and are often associated with perturbations in energy expenditure [4], [5].

Numerous epidemiological studies have suggested that exposure to persistent organic pollutants (POPs) contribute to the rising incidence of T2D [6]. For example, exposure to the organochlorine pesticide DDT has been found to be associated with increased odds of T2D [7]. Further, exposure to DDE, the primary metabolite of DDT, has also been associated with increased odds of excess body weight, insulin resistance, and dyslipidemia in people [8]. Because the World Health Organization still recommends the use of DDT for malaria control, DDT's potential contribution to the pathogenesis of T2D continues to be relevant to public health. Furthermore, obesity and T2D have increased at alarming rates in countries where DDT is still in use. For instance, the prevalence of diabetes among rural Indian adults increased 3.8 fold from 1989 to 2006 [9] and although a confluence of factors contribute to this rise, the contribution of endocrine disrupting chemicals cannot be ruled out without further study. Since epidemiological associations cannot establish causality, we tested whether exposure to DDT and DDE predisposes to T2D in mice at doses comparable to human exposures [10]–[13].

Both obesity and T2D are characterized by impaired energy expenditure and reduced thermogenesis, which are often the result of reduced brown adipose tissue (BAT) activity [5]. Mouse studies also suggest that impaired thermogenesis may precede insulin resistance [14]. Therefore, increasing energy expenditure by activating BAT thermogenesis may hold promise as a therapeutic strategy for managing both obesity and T2D [15]–[18].

Perturbations in the perinatal environment can impair metabolic programming that can consequently increase susceptibility to obesity and T2D in adulthood. A classic example of the importance of the perinatal environment on the future risk of T2D has been historically provided by the Dutch famine. Fetuses carried to term that were exposed to fetal malnutrition suffered from increased rates of the metabolic syndrome and glucose intolerance as adult offspring [19], [20]. The consequences of impaired fetal development on metabolic programming can be modeled in rodents, where fetal under-nutrition results in the development of the metabolic syndrome and glucose intolerance in adult offspring [21], [22]. Here we confirm the hypothesis that perinatal exposure to DDT impairs energy expenditure and metabolism leading to insulin resistance and the metabolic syndrome in adult offspring. We demonstrate for the first time that developmental exposure to DDT impairs BAT thermogenesis.

## Methods

### DDT exposure

To mimic the commercial formulation of DDT used as a pesticide in the US prior to its ban, 77.2% p,p′-DDT (98.5% purity neat, AccuStandard, New Haven, CT) and 22.8% o,p′-DDT (100% purity neat, AccuStandard) were dissolved at a final concentration of 0.17 g DDT mixture/L organic olive oil [23], hereafter referred to as DDT. We selected the dosing period to span liver and white- and brown- adipose tissue ontogenesis and programming [24]–[28]. From 11.5 days post coitus (determined by vaginal plug) to postnatal day (PND) 5, we administered 1.7 mg DDT/kg body weight (per os, n = 15 dams, 10 µL solution/g mouse), or equivalent volume of olive oil vehicle (hereafter referred to as vehicle or VEH, per os, n = 14 dams) daily to primigravid C57BL/6J mice (Jackson Laboratories, Bar Harbor, ME, Fig. S1). To determine the internal dose after transplacental and lactational transfer to pups [29], 4 additional dams were treated identically with DDT, and 24 hours after the final dose their sera was analyzed for o,p′-DDT, p,p′-DDT, and p,p′-DDE by gas chromatography using electron capture detection with detection limits of 0.36, 0.27 and 0.12 pg/µL serum respectively [30].

### Study Design

We culled litters to 6 pups after the final DDT dose on PND 5, to minimize infanticide common among C57BL/6J dams while normalizing lactational transfer of DDT and maternal behavior effects (Fig. S1). We evaluated timing of puberty daily by monitoring vaginal opening (VO) and preputial separation (PPS), in all female and male mice respectively, beginning at PND 18. On PND 21 we weaned pups (1 cage/sex/litter/treatment). We assessed body composition, energy homeostasis, metabolic parameters, and glucose tolerance up to 6 months of age when we randomized mice into 2 study arms to assess: 1) energy balance or 2) the metabolic consequences of high fat feeding (Fig. S1).

For the energy balance study, 10 females from 10 litters and 8 metabolic chambers were available. Energy balance was assessed by indirect calorimetry in 1 female mouse/litter (4 mice/perinatal treatment) for 7 days after 2 days of acclimation. Cold tolerance was assessed 3 days after indirect calorimetry in 5 female mice/perinatal treatment, as described below.

For the high fat feeding study, littermates were randomized to high fat diet (HFD, 4.73 kcal/g, 20% protein, 35% carbohydrate, and 45% fat per kcal; D12451, Research Diets, New Brunswick, NJ) or a low fat diet (LFD, 3.85 kcal/g, 20% protein, 70% carbohydrate, and 10% fat per kcal; D12450B, Research Diets) feeding following their glucose tolerance test (GTT) at 6 months of age for a total of 12 weeks (n = 8 mice/sex/litter per perinatal treatment and diet combination).

All mice had access to food and water *ad libitum* (other than during stated fasting periods) in sterile ventilated cages in an American Association for the Accreditation of Laboratory Animal Care-approved facility. At the end of all the studies, mice were sacrificed by exsanguination under isoflurane anesthesia. All animal experiments were conducted under the guidelines on humane use and care of laboratory animals for biomedical research published by the US National Institutes of Health. The procedures of this study were approved by the Mount Sinai School of Medicine Institutional Animal Care and Use Committee protocol #LA11-00116.

### Body composition

We measured body weight and percent fat mass by MRI (3in1-EchoMRI, Echo Medical Systems, Houston, TX) of each offspring monthly in 1–6 month old mice (n = 15 DDT- and n = 14 vehicle- exposed litters). The body mass of all mice was also measured at PND 5. During HF feeding, mice were weighed weekly and we used MRI to determine body composition at 8 months of age (n = 8 mice/sex/litter per perinatal treatment and diet combination).

### Energy homeostasis

We measured food intake in mice on a monthly basis from 2–6 months (n = 15 DDT- and n = 14 vehicle- exposed litters). In 2 month old mice, each mouse/sex/litter/treatment was singly housed and food intake was assessed daily over a 5 day period. In group- housed mice aged 3–6 months, we measured food intake per cage over a 2 day period. In the HF feeding study, we measured weekly food intake of singly housed mice fed LFD or HFD. Food intake (g or kcal) was calculated as the difference in food weight divided by the number of mice that had access to it and the number of days they had access, and transformed to kcal when comparing HFD and LFD (SAS 9.2, SAS Institute, Cary, NC). We measured core temperature monthly in all mice aged 2–5 and 8 months old by inserting a thermocouple into the rectum (BAT-10, Physitemp, Clifton, NJ).

For the energy balance studies, we housed 4 females/perinatal treatment (1 mouse/litter) individually in metabolic chambers, which continuously measured oxygen consumption, carbon dioxide production, food intake, and 3 plane-locomotor activity through open-circuit, indirect calorimetry (CLAMS, Columbus Instruments, Columbus, OH). During this 9 day period, we provided mouse powdered diet (NIH 07) and water *ad libitum* while they were maintained at 21–22°C for a 12-hour light/dark cycle. The respiratory exchange ratio (RER) was calculated as the quotient of vCO_2_/vO_2_.

Three days after the indirect calorimetry was completed, we individually housed 5 female mice/perinatal treatment (1 mouse/litter) at 4°C with bedding enrichment. Core body temperature was measured immediately upon exposure to 4°C, and every 30 minutes thereafter until 90 minutes, when 50% of the mice reached core temperatures of <30°C after being kept for 90-minutes at 4°C. Two days later, we housed each of the 10 female mice for 1 hour at 4°C prior to sacrifice.

### GTT

Glucose tolerance was assessed in 1 mouse/sex/litter/perinatal treatment aged 2, 3 and 6 months (randomized selection from n = 15 DDT- and n = 14 vehicle- exposed litters) and 1 mouse/sex/litter for each perinatal treatment and adult diet combination at eight months (randomized selection from n = 8 litters/treatment). Two-month-old mice received 2.0 mg D-glucose/g mouse body weight (i.p.), since at young age mice (as well as humans) are more glucose tolerant, and all other mice received 1.0 mg D-glucose/g mouse body weight (i.p.). For all GTT, we fasted mice for 5 hours, and collected sub-mandibular blood to determine fasting blood glucose (AlphaTrak, Abbott Laboratories, Abbott Park, IL) and fasting plasma metabolic parameters. We measured blood glucose 15, 30, 45, 60, 90, and 120 minutes after a D-glucose bolus for GTT. We determined total AUC using the trapezoidal approach (PROC SQL, SAS 9.4, SAS Institute, Cary, NC).

### Metabolic parameters

Trunk blood glucose was measured in all mice culled on PND 5. Sub-mandibular blood was collected from mice fasted for 5 hours (1 mouse/sex/litter/treatment) at ages 1–6 months old (randomized selection from n = 15 DDT- and n = 14 vehicle- exposed litters) and 8 months old (randomized selection from n = 8 litters/treatment) to determine blood glucose (AlphaTrak) and plasma insulin by ELISA (Mercodia, Uppsala, Sweden). Fasting plasma triglycerides, total cholesterol, and non-esterified fatty acids (NEFA) were evaluated enzymatically (Wako Chemicals, Richmond, VA). Homeostasis model assessment- insulin resistance (HOMA-IR) was calculated as the product of fasting glucose and insulin divided by 22.5 [31].

### Pathology

Livers were harvested from 1 female fed HFD per perinatal treatment group (randomized selection from n = 1 female/litter in 7 VEH and 8 DDT litters) into OCT medium, frozen 5 µM thick sections were stained with oil red O, and fields with representative staining of the sample and the least clear space (vessels, ducts, etc.) were selected. Histopathological evaluations were assessed by a veterinary pathologist who was blinded to the treatments.

### Hepatic lipids

Hepatic triglycerides and cholesterol esters were measured after a modified Folch extraction (randomized selection from n = 1 female/litter in 7 VEH and 8 DDT litters fed HFD) [32]. Total cholesterol [33] and triglycerides [34] were measured enzymatically from these livers (Fisher Diagnostics, Middletown, VA).

### Hepatic bile acids

A 15 mg liver sample (randomized selection from n = 1 female/litter in 7 VEH and 8 DDT litters fed HFD) was pulverized on dry ice, enriched with deuterated bile acid analogs, butylated hydroxytoluene and ethylinediaminetetraacetic acid, and extracted twice with 500 µL methanol. The combined extract was dried, reconstituted in 100 µL methanol:acetonitrile (50∶50, v/v) with internal standards 1-phenyl-3-hexanoic acid urea and 1-cyclohexyl-3-dodecanoic acid urea (Sigma-Aldrich, St. Louis, MO) and filtered through 0.1 µm filters. Tissue bile acids were isolated and quantified by LC-MS/MS using modifications of a previously published method [35]. Separation of bile acids was achieved using reversed phase UPLC with a 1.7 µm, 2.1×100 mm Acquity BEH C18 column (Waters, Milford, MA) using a 16 min gradient (Solvent A = 0.1% formic acid; Solvent B = 0.1% formic acid in acetonitrile) on a Waters Acquity UPLC (Waters) and detected on an API 4000 QTrap (AB Sciex, Framingham, MA, USA) by multiple reaction monitoring (MRM) after negative mode electrospray ionization. Residues were quantified against 7 point curves of authentic standards purchased from Steraloid Inc. (Newport, RI), Sigma-Aldrich (St. Louis, MO), and Medical Isotopes, Inc. (Pelham, NH) using internal standard methodologies.

### Western blot analysis

Protein expression was assessed as previously described [36]. Briefly, liver was homogenized and 10–20 µg of protein was subjected to Western blot analysis with primary antibodies against fatty acid synthase (FAS, BD Biosciences, San Jose, CA), phospho-acetyl-CoA carboxylase (ACC), ACC, phospho- adenosine triphosphate citrate lyase (ATPCL), ATPCL, adipose triglyceride lipase (ATGL), AKT Thr^308^ and Ser^473^, AKT, phospho- glycogen synthase kinase 3 (GSK3), GSK3, phospho-ERK, and ERK (all antibodies from Cell Signaling Technology, Danvers, MA unless stated otherwise) and insulin receptor (IR) beta (Santa Cruz Biotechnology, Dallas, TX). The secondary antibody was DyLight conjugated goat anti-rabbit or anti-mouse IgG (ThermoScientific). Signal intensity of proteins of interest was divided by the intensity of the housekeeping protein HSC 70 (Santa Cruz Biotechnology) to determine fold change (Odyssey, LI-COR, Lincoln, NE). We have confirmed the specificity of ATGL in adipose tissue specific ATGL knockout vs. wild type mice, and all other antibodies have been characterized and validated in prior peer review publications [(Table S1), [37]–[41]].

### Semi-quantitative PCR

mRNA was extracted with the RNeasy kit (Qiagen, Hilden, Germany) to synthesize cDNA using reverse transcription PCR (Applied Biosystems, Foster City, CA). Semi-quantitative PCR was performed using proprietary TaqMan primers for transcripts: peroxisome proliferative activated receptor gamma coactivator 1 alpha (*Ppargc1a*), iodothyronine type II deiodinase (*Dio2*), twist-related protein 1 (*Twist1*) and uncoupling protein 1 (*Ucp1*) in BAT using TATA box binding protein (*Tbp*) as an endogenous control; 3-hydroxy-3-methylglutaryl-Coenzyme A reductase (*Hmgcr*), low density lipoprotein receptor (*Ldlr*), and cytochrome P450 7a1 (*Cyp7a1*) in livers with glucuronidase beta (*Gusb*) as an endogenous control (Applied Biosystems). Additional semi-quantitative PCR was performed in BAT using SYBR Green with published primers: glucose transporter type 4 (*Glut4*, F: CATCCCACAAGGCACCCCTC, R: CATGCCACCCACAGAGAAGA [42]), lipoprotein lipase (*Lpl*, F:TGGAGAAGCCATCCGTGTG, R: TCATGCGAGCACTTCACCAG [42]), adipose triglyceride lipase (*Pnpla*, F: TGTTTCAGACGGAGAGAACGTC, R: TGAGAATGGGGACACTGTGATG [43]), and carnitine palmitoyltransferase 2 (*Cpt2*, F: CCAGCGGATAAACCACAACA, R: AAGCCACCGAGGCTCACTG [42]) using *Tbp* (F: GAAGCTGCGGTACAATTCCAG, R: CCCCTTGTACCCTTCACCAAT [44]) as endogenous control. Endogenous controls did not vary significantly across treatment groups. The 2^-ddCT^ method was used to approximate relative transcript fold change between treatment groups (coefficient of replicate variation <10%) [45].

### Statistical analyses

Phenotypes were assessed at specific ages by modeling the fixed effect of perinatal DDT and the random effect of litter while stratifying by gender (PROC MIXED, SAS). Longitudinal analyses of body mass, fat mass, and temperature were conducted using the age-specific model as described above, with the addition of age and an age*treatment term as fixed effects, and individual mouse identifiers as random effects. To assess circadian effects during indirect calorimetry, photoperiods and a photoperiod*perinatal treatment term were included as fixed effects in the age-specific model. Body weight and fat mass were considered as potential covariates in indirect calorimetry analyses [46] but because these body composition variables were not significantly different between perinatal treatments and caused no reduction in data significance or difference in interpretation, these covariates were not included in the final indirect calorimetry models. When examining phenotypes from the HF feeding study, diet and a diet*perinatal treatment term were included as fixed effects in the age-specific and longitudinal models described above. GTTs, fasting metabolic parameters, protein expression, mRNA, lipids, and bile acids were evaluated without random effects because only one mouse per litter was included (PROC GLM, SAS). To evaluate the potential covariate behavior of hepatic bile acids and other metabolic factors which may influence or be influenced by cholesterol metabolism, imDEV v 1.4.2 [47] was employed to normalize data, transform variables to mean centers with unit variance, and conduct partial least squares discriminate analysis (PLS DA; oscores pls). The dependent variable was the perinatal treatment group while the independent variables were hepatic bile acids, *Cyp7a1* fold-change, hepatic cholesterol and triglycerides, plasma cholesterol, % body fat, and age. Measures with variable importance in projection (VIP) scores >1 were considered important discriminators of treatment [48]. Timing of puberty was evaluated using survival analysis with treatment as a fixed effect and litter as a repeated event (PROC PHREG, SAS). In all models, we used a significance threshold of p<0.05 for main effects and p<0.1 for interaction effects.

## Results

### Perinatal DDT exposure increases adiposity in young adults

**Figure 1:**
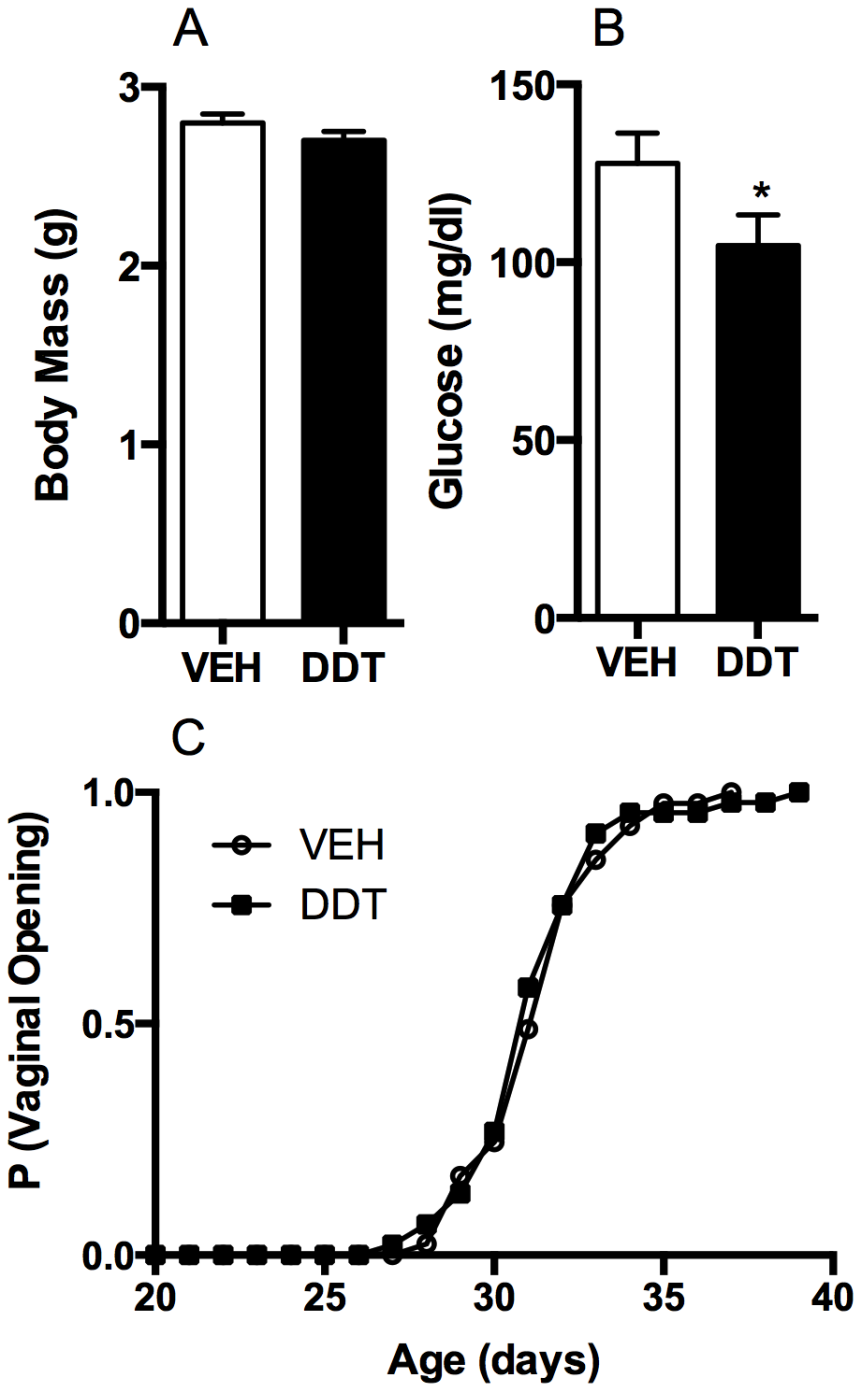
Effect of perinatal DDT on the development of female mice. (A) Female body mass at PND5 (n = 15 DDT- and n = 14 VEH- exposed litters). (B) Trunk blood glucose in PND5 females culled from litters >6 pups (n = 7 DDT- and n = 7 VEH- exposed litters). (C) Proportion of mice with vaginal opening as a function of age (n = 15 DDT- and n = 14 VEH- exposed litters). *p<0.05 DDT vs. vehicle controls. Data are represented as least squares (LS) means + SEM.

**Figure 2:**
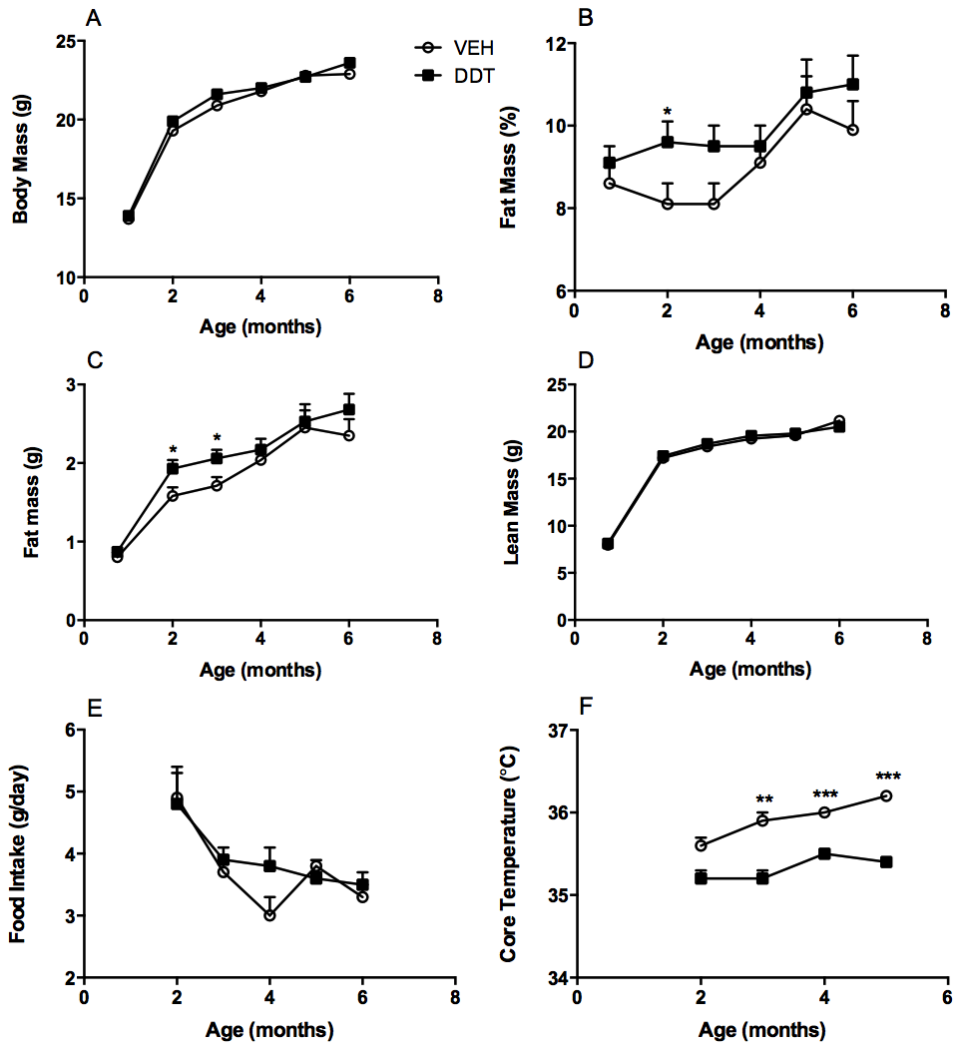
Effect of perinatal DDT on body composition and energy balance in female mice. Monthly (A) body mass (p = 0.05 at 2 months, p = 0.08 at 3 months), (B) percent adiposity (p = 0.05 at 3 months), (C) fat mass, (D) lean mass, and (E) food intake (A–E: n = 15 DDT- and n = 14 VEH- exposed litters). (F) Monthly core temperature (1 female/litter at 2 month time point, all females/litter thereafter, p = 0.07 at 2 months, age*DDT pi = 0.07). *p<0.05, **p<0.001, ***p<0.0001 DDT vs. vehicle controls. Data are represented as LS means + SEM.

To examine whether perinatal DDT exposure causes the metabolic syndrome in adult mouse offspring, dams were exposed to DDT from mid-gestation to PND 5. The resulting internal doses were 2.2 (±0.1) ng p,p′-DDE/mL, 51.1 (±10.2) ng p,p-DDT/mL, while o,p′-DDT was undetected [mean (± SEM)]. These exposures were sufficiently low as to have no impact on length of gestation, weight gain during gestation, or litter size (Fig. S2) and fall within the range of contemporary human exposures [10], [49]. Perinatal DDT exposure decreased body weight and blood glucose in male mice (Fig. S3A–B) and decreased glucose in female mice (Fig. 1B) on PND 5. Although DDT and DDE are ligands of the estrogen and androgen receptors [50], the timing of puberty in either sex was not significantly affected by the perinatal DDT exposure (Fig. 1C, Fig S3C). Perinatal DDT exposure caused a transient increase in body weight and adiposity of young adult female mice (Fig. 2A–C) but no differences in female lean mass (Fig. 2D), or male body composition were observed (Fig. S3D–E). Further, glucose tolerance, fasting glucose, insulin, and lipid levels were not altered by perinatal DDT exposure in either sex during their first 6 months of post-weaning life (Fig. S4, Table S2). These data suggest that mice exposed to perinatal DDT manifested only subtle features of the metabolic syndrome until adulthood. Since perinatal DDT exposure had no effect on food intake of offspring (Fig. 2E, Fig. S3F), the increased adiposity transiently observed among young female mice exposed to DDT could have resulted from impairments in thermogenesis and energy expenditure.

### Perinatal DDT exposure reduces energy expenditure

**Figure 3:**
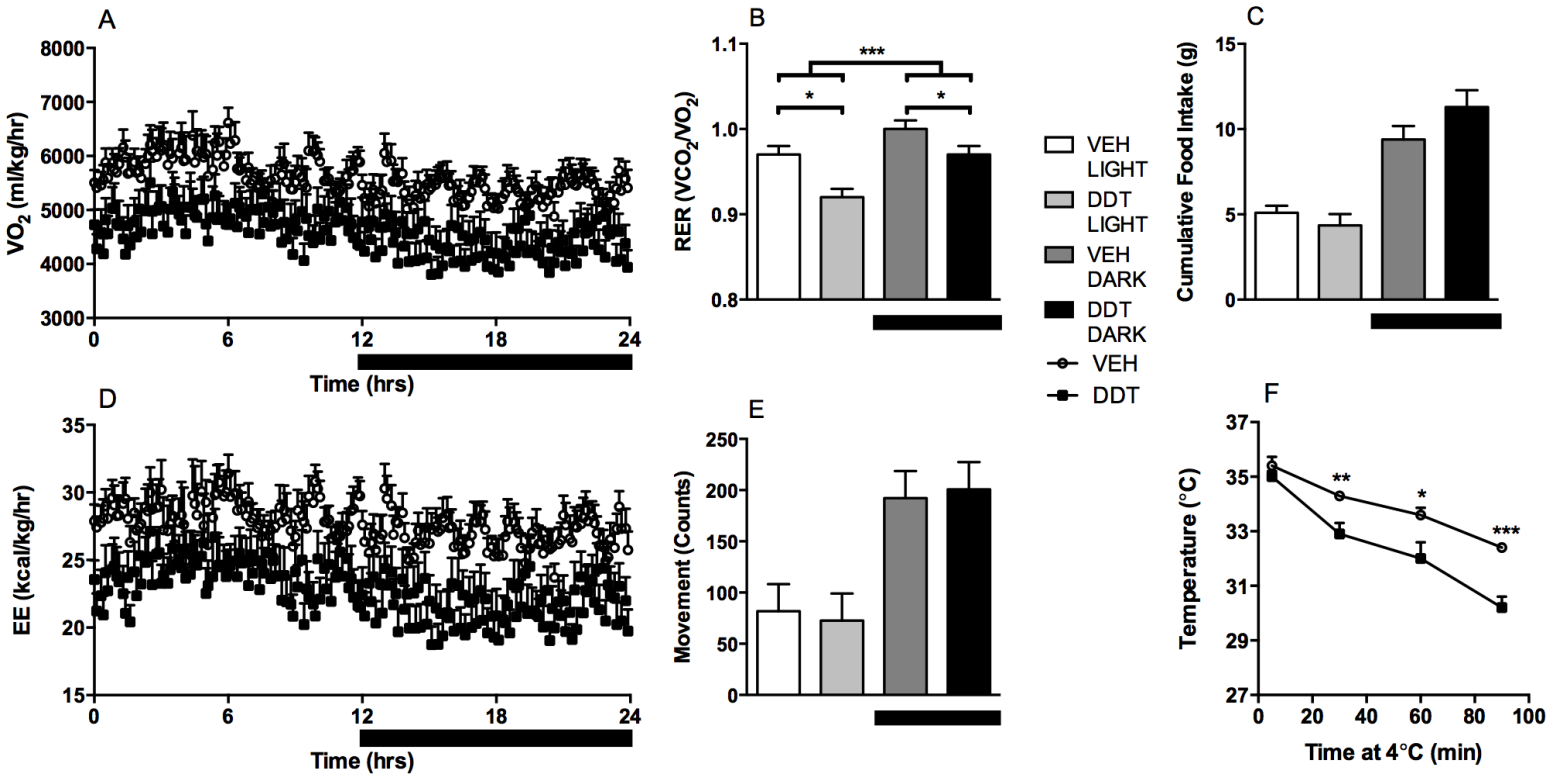
Effect of perinatal DDT on energy expenditure and cold tolerance in six month old female mice. (A) Oxygen consumption, (B) RER (period*DDT p_interaction_<0.0001), (C) cumulative food intake, (D) energy expenditure, and (E) movement (over 5 day period and 4 female/treatment, 1 mouse/litter). (F) Core temperature (5 females/treatment, 1 female/litter). *p<0.05, **p<0.01, ***p<0.001 DDT vs. vehicle controls. Data are represented as LS means + SEM.

To assess thermogenesis, core body temperature was measured longitudinally. Perinatal DDT exposure did not affect the core body temperature of male mice (Fig. S3G), while it decreased the core temperature of female mice, an effect that became more pronounced with age (Fig. 2F). At 6 months, female mice perinatally exposed to DDT had decreased oxygen consumption (Fig. 3A) and a more pronounced RER reduction in the light period than in the dark period, indicative of greater fasting lipid utilization (Fig. 3B), although food intake appeared equivocal across treatment groups (Fig. 3C). Female mice perinatally exposed to DDT also had decreased energy expenditure, while physical activity was not altered (Fig. 3D–E), suggesting that decreased energy expenditure was the result of decreased thermogenesis. To further establish that female mice perinatally exposed to DDT had compromised thermogenesis, we evaluated cold tolerance in female mice exposed to 4°C. Female mice with perinatal DDT exposure exhibited marked cold intolerance, necessitating the termination of the cold tolerance test after 90 minutes (Fig. 3F).

**Figure 4:**
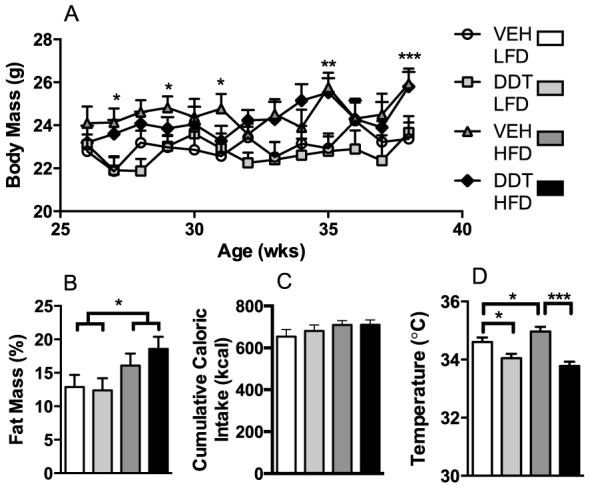
Effects of perinatal DDT and adult HFD feeding on body composition and energy balance in female mice (n = 1 female/litter in 7 VEH and 8 DDT litters). (A) Weekly body mass (HFD*age pi<0.0001). (B) Percent adiposity in 8 month old mice. (C) Cumulative caloric intake over 12 weeks of HFD or LFD feeding. (D) Core temperature in 8 month old mice (DDT*HFD pi = 0.01). *p<0.05, **p<0.01, ***p<0.0001 A–B) LFD vs. HFD only; D) significance between groups indicated by bars. Data are represented as LS means + SEM.

To determine if high fat feeding, a commonly used metabolic stressor to model human obesity, could unveil a metabolic phenotype caused by perinatal DDT exposure, mice were fed either LFD or HFD for 12 weeks beginning at 6 months of age. HF feeding increased caloric intake, adiposity, and body mass equally in all groups (Fig. 4A–C, Fig. S5), indicating that any metabolic alterations observed in HF fed mice that were perinatally exposed to DDT occurred independently of caloric intake and/or body composition.

Perinatal DDT exposure reduced core temperature in 9 month old female mice (Fig. 4D). HFD feeding led to higher body core temperatures in non-exposed mice, however this thermogenic adaptation to HFD was absent in mice perinatally exposed to DDT (Fig. 4D). Perinatal DDT exposure paradoxically reduced body core temperature by more than 1°C in mice fed HFD (p<0.0001). Thus, perinatal DDT impairs thermogenesis which is further worsened by HFD, indicating that developmental DDT exposure increases susceptibility to HFD induced- impairment in energy balance.

**Figure 5:**
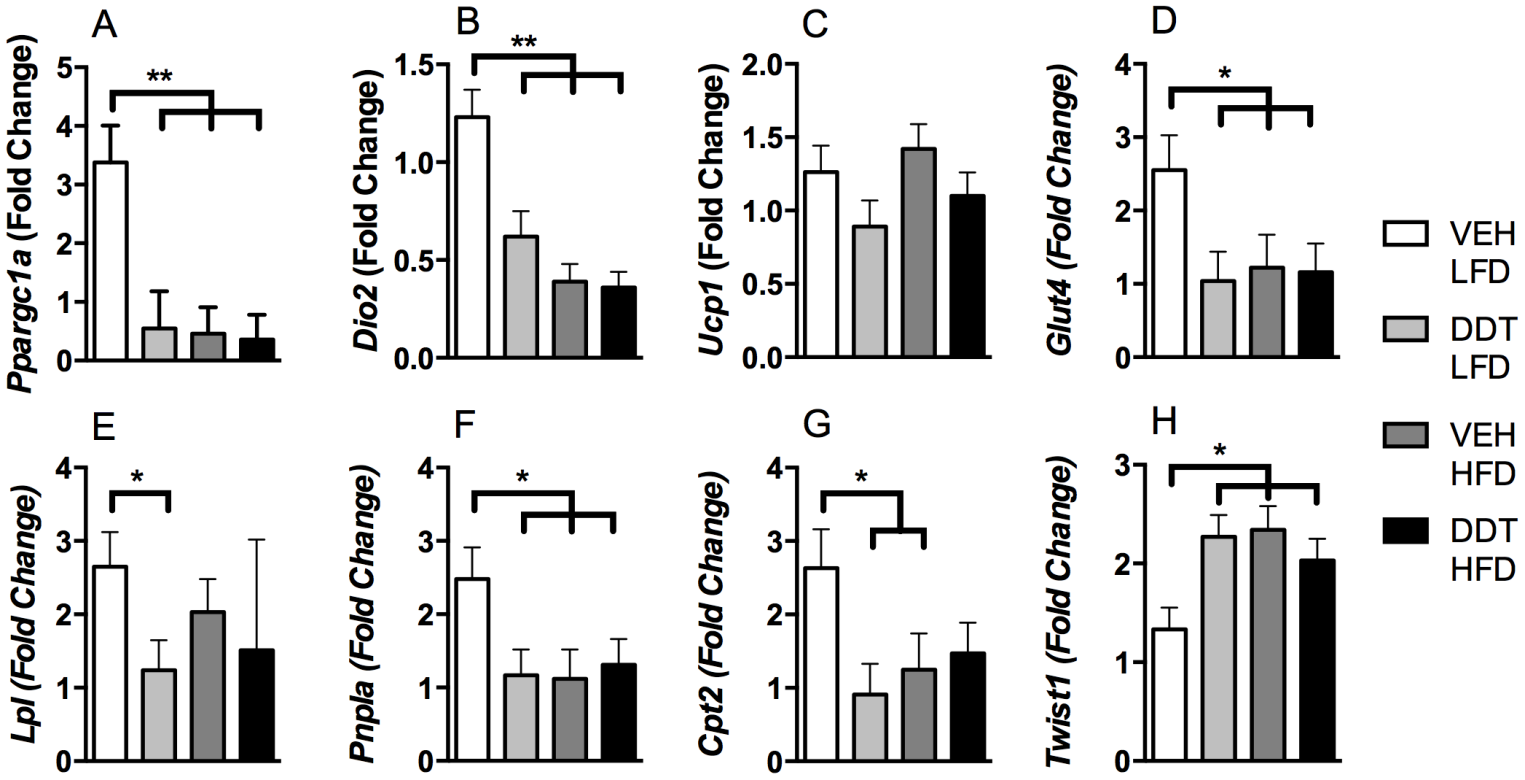
Effects of perinatal DDT and adult HFD feeding on RNA expression in BAT from 9 month old female mice (n = 1 female/litter in 8 VEH + LFD, 8 DDT + LFD, 7 VEH + HFD and 8 DDT + HFD litters). (A) *Ppargc1a*- (DDT*HFD pi<0.01), (B) *Dio2*- (DDT*HFD pi<0.01), (C) *Ucp1*-, (D) *Glut4*- (DDT*HFD pi<0.05), (E) *Lpl*- (DDT*HFD pi<0.05), (F) *Pnpla*- (DDT*HFD pi<0.05), (G) *Cpt2-* (DDT*HFD pi<0.05), and (H) *Twist1*- (DDT*HFD pi<0.1) fold change (relative to LFD + VEH and adjusted by *Tbp*). *p<0.05, **p<0.01 significance between groups indicated by bars. Data are represented as LS means + SEM.

We next examined if perinatal DDT exposure altered molecular mechanisms controlling thermogenesis in BAT of 9 month old female mice (Fig. 5). Compared to LFD-fed control mice, adult HF feeding and perinatal DDT exposure caused a reduction in mRNA expression of *Ppargc1a* (Fig. 5A), a master regulator of thermogenesis. Indeed HFD and DDT exposures reduced the expression of *Dio2* (Fig. 5B), which encodes an enzyme that locally activates thyroid hormone by deiodinating T4 to T3 to stimulate thermogenesis in BAT. However there was no significant change in *Ucp1* RNA expression (Fig. 5C), a protein that uncouples respiration to increase thermogenesis [51], [52]. Consistent with the regulatory role of PGC1A in substrate utilization [14], [53]–[56], perinatal DDT exposure and adult HF feeding decreased the expression of glucose transporter *Glut4*, lipoprotein transporter *Lpl* (no diet effect), lipolysis rate-limiting *Pnpla*, and the fatty acid oxidation shuttle *Cpt2* (Fig. 5D–G). Because transcription of *Ppargc1a* and its downstream targets was suppressed, we investigated negative regulation of *Ppargc1a* across DDT and HFD treatments. Indeed, expression of *Twist1*, a negative regulator of *Ppargc1a* [57], was increased in BAT of 9 month old mice exposed perinatally to DDT and fed a HFD during adulthood (Fig. 5H).

### Perinatal exposure to DDT increases susceptibility to HFD- induced insulin resistance

**Figure 6:**
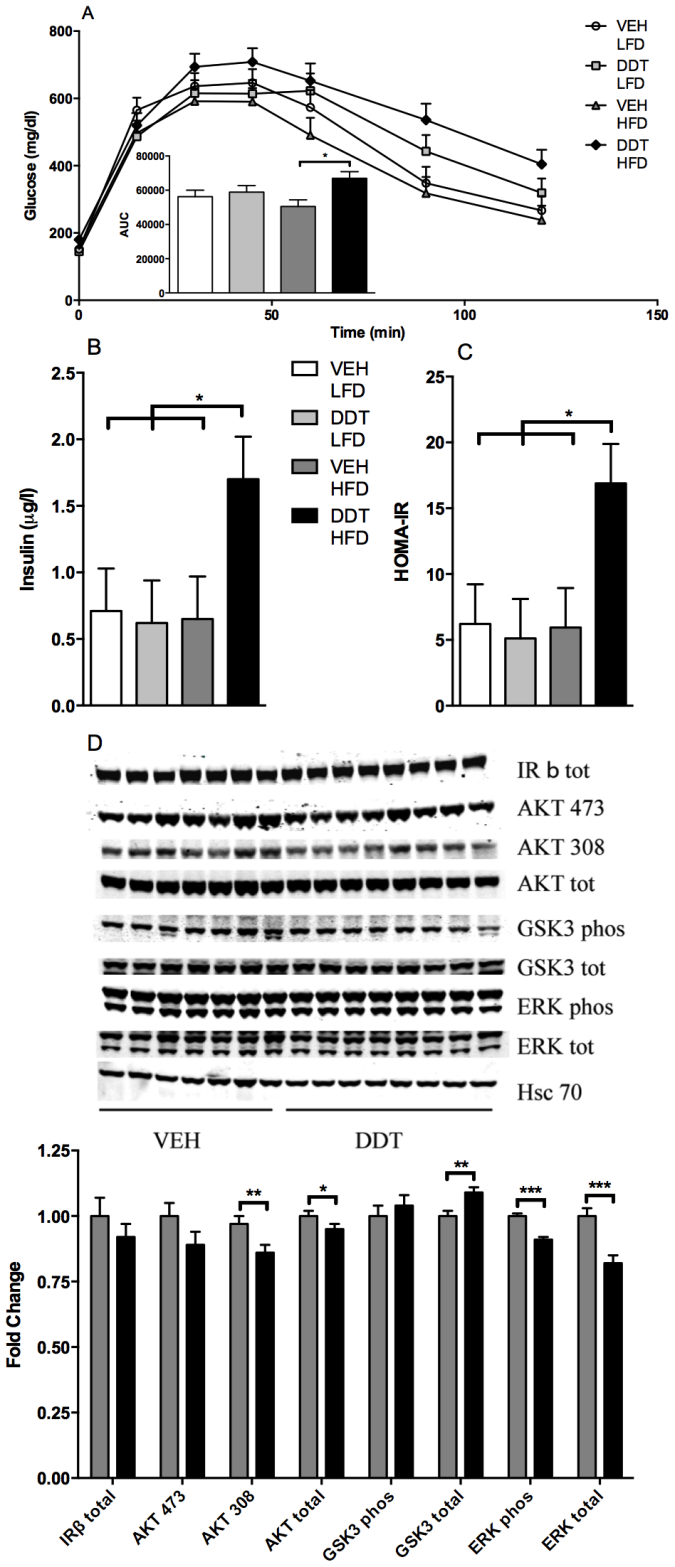
Effects of perinatal DDT and adult HFD feeding on glucose homeostasis in adult female mice (n = 1 female/litter in 8 VEH + LFD, 8 DDT + LFD, 7 VEH + HFD and 8 DDT + HFD litters). (A) Glucose tolerance test and resulting AUC (DDT*HFD pi = 0.08), (B) fasting insulin (DDT*HFD pi = 0.08) and (C) HOMA-IR (DDT*HFD pi = 0.07) in 8 month old mice. (D) Hepatic IRβ total, AKT 473 phosphorylation, AKT 308 phosphorylation, AKT total, GSK 3 phosphorylation, GSK 3 total, ERK phosphorylation, and ERK total fold change (relative to LFD + VEH and adjusted by HSC 70) as assessed by Western blot analysis (n = 1 female/litter in 7 VEH and 8 DDT litters) in 9 month old mice fed HFD. *p<0.05, **p<0.01, ***p<0.001 significance between groups indicated by bars. Data are represented as LS means + SEM.

Female mice maintained on a LFD and exposed perinatally to DDT had equivocal glucose tolerance compared to vehicle treated controls (Fig. 6A). However, after HF feeding for 12 weeks, female mice exposed perinatally to DDT developed glucose intolerance and insulin resistance as evidenced by higher AUC during GTT, fasting hyperinsulinemia, and increased HOMA-IR (Fig. 6A–C). Of note, this constellation is often observed in prediabetic patients that commonly have the metabolic syndrome. Further, the expression and activation states of AKT and ERK, proteins involved in insulin signaling, were moderately decreased in female mice perinatally exposed to DDT and fed a HFD (Fig. 6D–E), although it remains unclear how much these moderate decreases in hepatic insulin signaling contribute to the observed glucose intolerance and insulin resistance. However, male mice perinatally exposed to DDT developed glucose intolerance when fed LFD, but not HFD (Fig. S8A–B). Thus, perinatal exposure to DDT increases the susceptibility to HFD- induced glucose intolerance in female but not male mice (Fig. 6, Fig. S6). The sex differences will require further studies in the future to test if this is estrogen dependent, but of note, female mice (and humans) are somewhat protected from HF feeding- induced glucose intolerance [58].

### Perinatal DDT exposure and high fat feeding cause mild dyslipidemia in female mice

**Figure 7:**
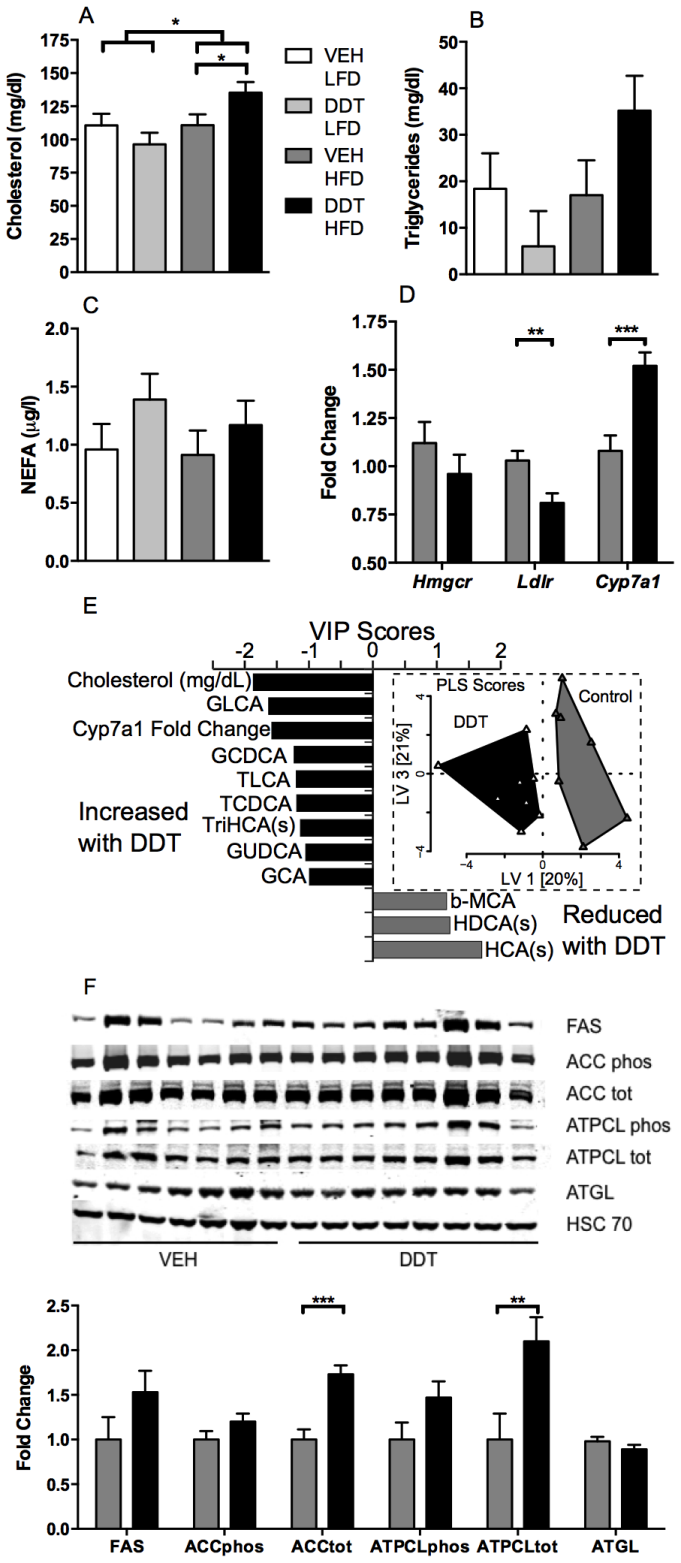
Effects of perinatal DDT and adult HFD feeding on lipid homeostasis in adult female mice (n = 1 female/litter in 8 VEH + LFD, 8 DDT + LFD, 7 VEH + HFD and 8 DDT + HFD litters). Fasting- (A) total cholesterol (HFD*DDT pi = 0.02), (B) triglycerides (HFD*DDT pi = 0.16), and (C) NEFA in 8 month old mice. (D) Hepatic *Hmgcr*-, *Ldlr*-, and *Cyp7a1*- fold change (relative to LFD + VEH and adjusted by *Gusb*) in 9 month old female mice fed HFD. (E) Bile acid metabolites discriminated by DDT or VEH exposure (VIP>1). Inset: The PLS-DA Scores Plot demonstrates clear discriminatory separation of mice with or without perinatal DDT exposure along latent variable 1 (LV1). Symbols represent individual animals. Metabolite abbreviations followed by “(s)” indicate “screening” data without the benefit of authentic calibration standards. F) Hepatic FAS- (p = 0.14), ACC phosphorylation - (p = 0.13), ACC total-, ATPCL phosphorylation - (p = 0.07), ATPCL total-, and ATGL- (p = 0.22) fold change (relative to LFD + VEH and adjusted by HSC 70) in 9 month old female mice fed HFD. *p<0.05, **p<0.01, ***p<0.001 significance between groups indicated by bars. Data are represented as LS means + SEM.

To evaluate whether perinatal DDT exposure impaired lipid metabolism in adult HFD fed- mice, we measured fasting triglycerides, cholesterol, and NEFAs (Fig. 7, Fig. S6). Perinatal DDT exposure significantly increased fasting cholesterol in female mice fed HFD, although it had a negligible effect on cholesterol in female mice fed LFD (Fig. 7A). A similar, but non-significant trend was observed in fasting triglycerides levels in female mice (Fig. 7B), whereas NEFAs were not altered by perinatal DDT or HFD in female mice (Fig. 7C). Thus perinatal DDT exposure combined with HFD caused mild dyslipidemia in female (Fig. 7A) but not male mice (Fig. S6E-G). We also examined whether perinatal DDT exposure altered the expression of mRNA and proteins associated with the regulation of lipid homeostasis in livers from female mice fed HFD. mRNA expression of *Ldlr*, a cholesterol transporter, was significantly decreased, whereas that of *Cyp7a1*, the rate limiting enzyme in the synthesis of bile acids from cholesterol, was significantly increased by perinatal DDT exposure (Fig. 7D). Changes in hepatic bile acid metabolism among females fed HFD were consistent with these changes in *Cyp7a1* expression due to perinatal DDT exposure. Specifically, conjugated bile acids, including GLCA, TLCA, and TDCA, and the C27 precursor TriHCA, increased with DDT treatment and these conjugated bile acid changes were closely correlated with hepatic *Cyp7a1* expression and plasma cholesterol (VIP>1, Fig. 7E, Fig. S7, Table S3). Conversely, unconjugated bile acids, including HCA, HDCA, and β-MCA, were relatively lower in the DDT exposed versus control groups and were closely associated with hepatic lipids (VIP>1, Fig. 7E, Fig. S7, Table S3). Hepatic protein expression of two lipogenic enzymes ACC and ATPCL was also increased, consistent with increased hepatic lipogenesis as a consequence of perinatal DDT exposure (Fig. 7F). This did not appear to contribute to steatosis, as hepatic lipid content was not increased due to perinatal DDT exposure among female mice fed HFD (Fig. S8).

## Discussion

Here we investigated the hypothesis that perinatal DDT exposure impairs energy expenditure and metabolism in adult mice. Our results suggest that perinatal DDT exposure decreases energy expenditure, and impairs the regulation of thermogenesis, glucose-, and lipid- homeostasis, which contribute to the development of insulin resistance and the metabolic syndrome in adult female mice.

### Obesity and thermogenesis

Perinatal DDT exposure increased adiposity in young adult female mice. This transient increase in adiposity may have resulted from the profoundly impaired thermogenesis we observed after DDT exposure, which was greater than what was observed in UCP1 knock-out mice [59]. Impaired thermogenesis may have been a consequence of reduced energy supply and utilization in BAT as perinatal DDT exposure significantly decreased expression of lipolytic *Pnpla* and substrate transporters (*Glut4*, *Lpl*, *Cpt2*) [14], [53]–[56]. Because food intake and physical activity were equal among groups, the reduced energy expenditure would be predicted to increase fat mass more profoundly than what we observed. Whether increased caloric content in feces accounts for this discrepancy will require further study [60].

### Insulin resistance and thermogenesis

Epidemiological studies have demonstrated associations between adult DDT and DDE exposures and diabetes in humans [7], [8], [61], [62]. Similarly, adult rodents exposed to high doses of DDT had impaired insulin secretion, glucose intolerance, and elevated gluconeogenesis [63], [64]. Here we find that female mice perinatally exposed to low doses of DDT and subsequently fed a HFD in adulthood developed impaired glucose tolerance, increased fasting insulin, and HOMA-IR, indicative of insulin resistance and increased risk for T2D. We have observed that *Ppargc1a* expression in BAT of adult female offspring was reduced after perinatal DDT exposure. *Ppargc1a* plays an important role linking thermogenesis to risk of T2D as illustrated by genetic ablation studies of *Ppargc1a* in mice, after which thermogenesis was impaired and susceptibility to HFD-induced insulin resistance, but not obesity, was increased [14]. Whether the reduced *Ppargc1a* expression in BAT of DDT-exposed mice accounts for their reduced thermogenesis and insulin resistance needs further study. It is plausible that the effects of perinatal DDT on thermogenesis and insulin resistance in mice may be relevant to humans who suffered elevated DDT exposure perinatally.

### Lipid homeostasis and adaptive energy metabolism

We also observed that perinatal DDT exposure caused a modest increase in circulating lipids after HFD feeding, which may in part be due to increased hepatic de novo lipogenesis as suggested by the increased protein expression of key lipogenic enzymes in the liver. Previous studies suggest that acute DDT administration increases hepatic cholesterol and triglyceride synthesis, resulting in their increased circulation in both rats and monkeys [65], [66]. It is plausible that DDT and HFD exposures caused higher hepatic de novo lipogenesis, which was compensated for by increased lipid utilization, as evidenced by reduced RER, and increased hepatic bile acid conjugation, to prevent the development of hepatic steatosis in the female mice here [67], [68].

### Developmental origins of metabolic disruption

Several lines of evidence implicate developmental origins in DDT-induced metabolic syndrome. First, when the developmental stage of newborn mice was equivalent to that of a human fetus at term, pups exposed to DDT exhibited reduced body weights and glucose levels. This may contribute to their increased susceptibility to the metabolic syndrome since human studies have demonstrated that low birth weight and low neonatal glucose can be associated with the metabolic syndrome in adulthood [20], [69], [70]. Second, perinatal DDT exposure was associated with early-adult obesity in female offspring here and across generations in other rodent studies [71], [72]. While sex differences are common in mouse models of metabolic disease [73], and both DDT and DDE have estrogenic and anti-androgenic properties [50] the basis for the sex differences observed in our study will have to be delineated in future studies. Further, these data corroborate a prospective study of women where higher cord blood levels of DDE were associated with significantly increased BMI of the adult daughters in a dose dependent manner [74].

### Conclusions

Our study is the first to establish a link between developmental exposure to DDT and a moderately increased risk for the metabolic syndrome in adult offspring. Further, we know of no other studies implicating impaired thermogenesis in toxicant-induced insulin resistance and the metabolic syndrome. Given that circulating DDT and DDE levels in our mice fell within the range of DDT and DDE found in serum of contemporary people living in northern Europe and in malaria-infested regions, and of the pregnant mothers of US adults who are currently in their mid-fifties [7], [49], [75], [76] this is an important observation. Thus, it is likely that perinatal DDT exposure represents a risk factor for reduced energy expenditure in adult humans, even decades after DDT usage had ceased. The exact mechanism of this metabolic toxicity is unknown and how much it contributes to insulin resistance and dyslipidemia in the current obesity and diabetes epidemic will require further studies. But even small dysmetabolic effects of DDT and DDE are likely to be important from a public health perspective due to the ubiquity of these exposures among aging US and European populations as well as current populations of some developing and circumpolar countries. Given that the ban of DDT has reduced DDT exposure among the US population by about 100 fold [77], replacing DDT in malaria control should decrease DDT exposures over time and may reduce the susceptibility to the metabolic syndrome in future generations.

## Supporting Information

Figure S1 **Schematic of study design.** 1.7 mg DDT/kg body weight was administered to dams daily on gestational day 11 through postnatal day 5 and litters were culled to 6 mice at postnatal day 6. Mice were metabolically screened from 2–6 months when they were randomized into 2 independent study arms: energy balance and HFD or LFD feeding. Indirect calorimetry and cold tolerance were assessed in 6-month-old mice. Metabolic parameters (e.g. body composition, fasting lipids, glucose, and insulin, GTT) were assessed during 12 weeks of HFD or LFD feeding until mice were sacrifice at 9 months of age.
(DOCX)

Figure S2 **Effect of prenatal DDT on gestation.** (A) Gestation length, (B) gestation weight gain, and (C) litter size (n = 15 DDT litters and n = 14 VEH litters). Data are represented as LS means ± SEM.
(DOCX)

Figure S3 **Effect of perinatal DDT on body composition and energy balance in male mice.** (A) Body mass at PND5 (n = 15 DDT- and n = 14 vehicle- exposed litters). (B) Trunk glucose in PND5 males culled from litters >6 pups (n = 7 DDT and n = 7 VEH litters). (C) Proportion of males with preputial separation as a function of age (n = 15 DDT and n = 14 VEH litters). Monthly (D) body mass and (E) percent adiposity, (F) food intake, and (G) rectal temperature ((n = 15 DDT and n = 14 VEH litters, 1 mouse/litter at 2 month time point, all males/litter thereafter). Data are represented as LS means ± SEM. *p<0.05 DDT vs. vehicle controls.
(DOCX)

Figure S4 **Effects of perinatal DDT on glucose tolerance.** GTT in (A) female and (B) male 2 month old mice (2.0 mg glucose/kg body mass, 1.5 mg glucose/kg body mass, n = 15 DDT-exposed litters and n = 14 oil-exposed litters, 1 mouse/litter), in (C) female and (D) male 3 month old mice (1.0 mg glucose/kg body mass, 1.5 mg glucose/kg body mass, n = 12 litters/treatment, 1 mouse/litter), and in (E) female and (F) male 6 month old mice (1.0 mg glucose/kg body mass, n = 15 DDT-exposed litters and n = 14 oil-exposed litters, 1 mouse/litter). Data are represented as LS means ± SEM.
(DOCX)

Figure S5 **Joint effects of perinatal DDT and HFD on body composition and energy balance in male mice.** (A) Weekly body mass (n = 8 litters/treatment, 1 mouse/litter, HFD*age pi<0.0001). (B) Percent adiposity in 8 month old mice (n = 8 litters/treatment, 1 mouse/litter). (C) Cumulative food intake over 12 week HFD and LFD feeding period (n = 8 litters/treatment, 1 mouse/litter). (D) Rectal temperature in 8 month old mice (n = 8 litters/treatment, 1 mouse/litter). *p<0.05, **p<0.01, ***p<0.0001 LFD vs. HFD. Data are represented as LS means ± SEM.
(DOCX)

Figure S6 **Joint effects of perinatal DDT and HFD on glucose and lipid homeostasis in male mice.** (A) Glucose tolerance test at 1 mg glucose/kg body mass and (B) corresponding AUC, in 8 month old mice (n = 8 litters/treatment, 1 mouse/litter). (C) Insulin, (D) HOMA-IR, (E) total cholesterol, (F) triglycerides, and (G) NEFA (μg/l plasma) in 8 month old mice fasted for 5 hrs (n = 8 litters/treatment, 1 mouse/litter). LFD vs. HFD. *p<0.05 significance between groups indicated by bars. Data are represented as LS means ± SEM.
(DOCX)

Figure S7 **Partial Least Squares Discrimination Analysis (PLS-DA) of hepatic bile acids, triglycerides, and other metabolic factors which may influence or be influenced by cholesterol metabolism segregate treatment groups.** (B) Relative weight of independent variable to this segregation are shown in the PLS Loadings Plot. Hierarchical cluster analyses were used to segregate variables and 4 unique clusters are displayed with unique colors. Variables with the variable importance in projection VIP scores >0.8 or <−0.8 are labeled, >1 or <−1 shown as triangles. (C) All LV1 VIP scores. Metabolite abbreviations followed by “(s)” indicate “screening” data relative areas generated from the expected precursor > product ion mass transition in the LC-MS/MS system, without the benefit of authentic calibration standards.
(DOCX)

Figure S8 **Lipid content of adult female mice fed HFD.** (A) Oil red O stained liver from 9 month old female mice fed HFD for 12 weeks with exposure to perinatal vehicle (top) or DDT (bottom, bar  = 50 microns). (B) Hepatic triglycerides and cholesterol in 9 month old female mice fed HFD. Data are represented as LS means + SEM.
(DOCX)

Table S1 **Table of Information about Western Blot Antibodies.**
(DOCX)

Table S2 **Effects of perinatal DDT on metabolic parameters in mice fed regular chow.** LS Means (± SEM) shown.
(DOCX)

Table S3 **Liver Bile Acid concentrations (nmol/g).** Values are reported as mean ± SEM).
(DOCX)
