# Correction: Perinatal Exposure of Mice to the Pesticide DDT Impairs Energy Expenditure and Metabolism in Adult Female Offspring

**DOI:** 10.1371/journal.pone.0107332

**Published:** 2014-09-02

**Authors:** The PLOS ONE Staff

There is an error in the corresponding author designations. Christoph Buettner should be listed as a co-corresponding author. The correct corresponding authors are:

Michele La Merrill mlamerrill@ucdavis.edu

Christoph Buettner christoph.buettner@mssm.edu

There are errors in the Author Contributions. Christoph Buettner should be listed as one of the persons who conceived and designed the experiments, analyzed the data, contributed reagents/materials/analysis tools and contributed to the writing of the manuscript. The correct contributions are:

Conceived and designed the experiments: MLM and CB. Performed the experiments: MLM EK CL MLF. Analyzed the data: MLM EK EM CL MLF JN and CB. Contributed reagents/materials/analysis tools: MLM JN CB. Contributed to the writing of the manuscript: MLM EK EM CL MLF JN CB.
