# Supplementary figures and images for: Perinatal Exposure of Mice to the Pesticide DDT Impairs Energy Expenditure and Metabolism in Adult Female Offspring
Source: PLoS One. 2014 Jul 30;9(7):e103337. doi: 10.1371/journal.pone.0103337 (PMC4116186; doi:10.1371/journal.pone.0103337)

| 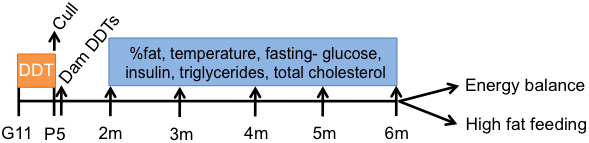 |
| --- |
|  |

Supplement: Figure S1 — Schematic of study design. 1.7 mg DDT/kg body weight was administered to dams daily on gestational day 11 through postnatal day 5 and litters were culled to 6 mice at postnatal day 6. Mice were metabolically screened from 2–6 months when they were randomized into 2 independent study arms: energy balance and HFD or LFD feeding. Indirect calorimetry and cold tolerance were assessed in 6-month-old mice. Metabolic parameters (e.g. body composition, fasting lipids, glucose, and insulin, GTT) were assessed during 12 weeks of HFD or LFD feeding until mice were sacrifice at 9 months of age. (DOCX) [file pone.0103337.s001.docx]

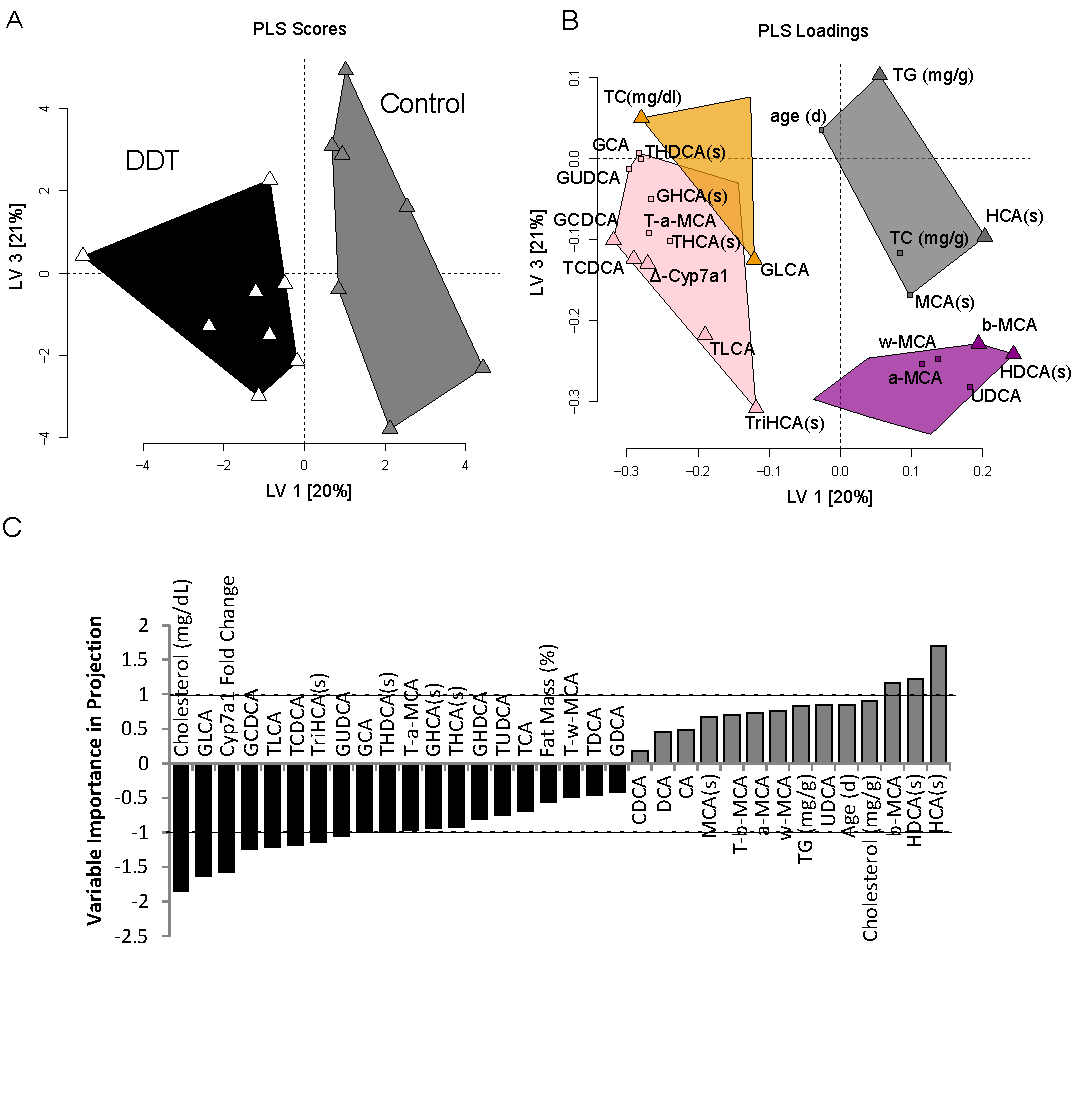

Supplement: Figure S7 — Partial Least Squares Discrimination Analysis (PLS-DA) of hepatic bile acids, triglycerides, and other metabolic factors which may influence or be influenced by cholesterol metabolism segregate treatment groups. (B) Relative weight of independent variable to this segregation are shown in the PLS Loadings Plot. Hierarchical cluster analyses were used to segregate variables and 4 unique clusters are displayed with unique colors. Variables with the variable importance in projection VIP scores >0.8 or <−0.8 are labeled, >1 or <−1 shown as triangles. (C) All LV1 VIP scores. Metabolite abbreviations followed by “(s)” indicate “screening” data relative areas generated from the expected precursor > product ion mass transition in the LC-MS/MS system, without the benefit of authentic calibration standards. (DOCX) [file pone.0103337.s007.docx]
