# Supplementary material for: Perinatal Exposure of Mice to the Pesticide DDT Impairs Energy Expenditure and Metabolism in Adult Female Offspring
Source: PLoS One. 2014 Jul 30;9(7):e103337. doi: 10.1371/journal.pone.0103337 (PMC4116186; doi:10.1371/journal.pone.0103337)
Supplement: Figure S5 — Joint effects of perinatal DDT and HFD on body composition and energy balance in male mice. (A) Weekly body mass (n = 8 litters/treatment, 1 mouse/litter, HFD*age pi<0.0001). (B) Percent adiposity in 8 month old mice (n = 8 litters/treatment, 1 mouse/litter). (C) Cumulative food intake over 12 week HFD and LFD feeding period (n = 8 litters/treatment, 1 mouse/litter). (D) Rectal temperature in 8 month old mice (n = 8 litters/treatment, 1 mouse/litter). *p<0.05, **p<0.01, ***p<0.0001 LFD vs. HFD. Data are represented as LS means ± SEM. (DOCX) [file pone.0103337.s005.docx]

|  |
| --- |
