# Supplementary material for: Perinatal Exposure of Mice to the Pesticide DDT Impairs Energy Expenditure and Metabolism in Adult Female Offspring
Source: PLoS One. 2014 Jul 30;9(7):e103337. doi: 10.1371/journal.pone.0103337 (PMC4116186; doi:10.1371/journal.pone.0103337)
Supplement: Table S1 — Table of Information about Western Blot Antibodies. (DOCX) [file pone.0103337.s009.docx]

| **Antibody** | **Company** | **CAT no.** | **Species** | **Mono/polyclonal** | **Dilution** |
| --- | --- | --- | --- | --- | --- |
| **FAS** | BD Bioscience | 610962 | Mouse | Monoclonal | 1:500 |
| **pACC** | Cell Signaling | 3661 | Rabbit | Polyclonal | 1:1000 |
| **ACC** | Cell Signaling | 3662 | Rabbit | Polyclonal | 1:1000 |
| **pATPCL** | Cell Signaling | 4331 | Rabbit | Polyclonal | 1:1000 |
| **ATPCL** | Cell Signaling | 4332 | Rabbit | Polyclonal | 1:1000 |
| **ATGL** | Cell Signaling | 2138 | Rabbit | Polyclonal | 1:1000 |
| **Akt 473** | Cell Signaling | 4058 | Rabbit | Monoclonal | 1:1000 |
| **Akt 308** | Cell Signaling | 2965 | Rabbit | Monoclonal | 1:1000 |
| **AKT** | Cell Signaling | 9272 | Rabbit | Polyclonal | 1:1000 |
| **pGsk3** | Cell Signaling | 9331 | Rabbit | Polyclonal | 1:1000 |
| **Gsk3 beta** | Cell Signaling | 9315 | Rabbit | Monoclonal | 1:1000 |
| **pERK** | Cell Signaling | 9101 | Rabbit | Polyclonal | 1:1000 |
| **ERK** | Cell Signaling | 9102 | Rabbit | Polyclonal | 1:1000 |
| **IR** | Santa Cruz | sc-711 | Rabbit | Polyclonal | 1:500 |
| **HSC70** | Santa Cruz | sc-7298 | Mouse | Monoclonal | 1:2000 |
