# Supplementary material for: Perinatal Exposure of Mice to the Pesticide DDT Impairs Energy Expenditure and Metabolism in Adult Female Offspring
Source: PLoS One. 2014 Jul 30;9(7):e103337. doi: 10.1371/journal.pone.0103337 (PMC4116186; doi:10.1371/journal.pone.0103337)
Supplement: Table S2 — Effects of perinatal DDT on metabolic parameters in mice fed regular chow. LS Means (± SEM) shown. (DOCX) [file pone.0103337.s010.docx]

| Metabolic Parameter | Age (months) | Female | |  | Male | |
| --- | --- | --- | --- | --- | --- | --- |
|  |  | Vehicle | DDT |  | Vehicle | DDT |
| Glucose (mg/dl) | |  |  |  |  |  |
|  | 0.75 | 176.2 (3.7) | 184.8 (3.5) |  | 207.8 (3.9) | 203.2 (4.0) |
|  | 2 | 161.9 (4.2) | 159.8 (4.0) |  | 187.6 (8.2) | 180.6 (7.9) |
|  | 3 | 156.5 (5.9) | 154.5 (5.4) |  | 171.1 (4.9) | 159.9 (4.7) |
|  | 4 | 157.4 (3.4) | 158.9 (3.2) |  | 186.3 (6.4) | 177.6 (5.7) |
|  | 5 | 157.0 (2.4) | 160.1 (2.3) |  | 199.4 (4.4) | 181.6 (4.2) |
|  | 6 | 143.4 (4.5) | 135.4 (4.3) |  | 165.0 (8.4) | 156.5 (8.1) |
| Insulin (μg/l) | |  |  |  |  |  |
|  | 2 | 0.71 (0.10) | 0.74 (0.13) |  | 1.23 (0.19) | 1.38 (0.17) |
|  | 3 |  |  |  | 1.31 (0.14) | 1.09 (0.13) |
|  | 4 | 0.40 (0.12) | 0.60 (0.10) |  | 1.05 (0.17) | 1.30 (0.15) |
|  | 5 | 0.53 (0.03) | 0.58 (0.05) |  | 1.35 (0.23) | 1.80 (0.22) |
|  | 6 | 3.21 (0.64) | 3.34 (0.54) |  | 1.36 (0.20) | 1.79 (0.19) |
| Triglycerides (mg/dl) | |  |  |  |  |  |
|  | 2 | 27.9 (2.7) | 32.4 (2.5) |  | 38.3 (8.2) | 54.0 (7.7) |
|  | 3 | 25.9 (3.1) | 24.9 (3.0) |  | 44.1 (7.1) | 48.2 (6.8) |
|  | 4 | 34.6 (0.6) | 37.9 (0.4) |  | 58.3 (5.1) | 55.4 (5.3) |
|  | 5 | 29.8 (1.8) | 32.9 (1.7) |  | 53.8 (5.2) | 59.6 (5.0) |
|  | 6 | 30.2 (3.2) | 32.1 (3.0) |  | 44.5 (3.4) | 58.2 (3.2) |
| Total cholesterol (mg/dl) | | |  |  |  |  |
|  | 2 | 97.6 (3.9) | 90.6 (3.7) |  | 184.7 (8.3) | 181.7 (7.7) |
|  | 3 | 111.1 (3.8) | 108.4 (3.5) |  | 120.1 (6.5) | 133.0 (6.5) |
|  | 4 | 74.1 (2.9) | 67.7 (2.8) |  | 100.1 (4.8) | 89.8 (4.5) |
|  | 5 | 74.6 (2.6) | 77.4 (2.5) |  | 105.0 (2.9) | 103.5 (2.8) |
|  | 6 | 105.2 (3.5) | 98.1 (3.4) |  | 113.5 (4.5) | 105.4 (4.4) |
