# Supplementary material for: Perinatal Exposure of Mice to the Pesticide DDT Impairs Energy Expenditure and Metabolism in Adult Female Offspring
Source: PLoS One. 2014 Jul 30;9(7):e103337. doi: 10.1371/journal.pone.0103337 (PMC4116186; doi:10.1371/journal.pone.0103337)
Supplement: Table S3 — Liver Bile Acid concentrations (nmol/g). Values are reported as mean ± SEM). (DOCX) [file pone.0103337.s011.docx]

| **Unconjugated Bile Acids** | |  | | **DDT** | **Control** |
| --- | --- | --- | --- | --- | --- |
| Cholic acid | | CA | | 147 ±47 | 205 ±83 |
| Chenodeoxycholic acid | | CDCA | | 0.111 ±0.03 | 0.158 ±0.088 |
| Deoxycholic acid | | DCA | | 0.368 ±0.086 | 0.691 ±0.33 |
| Ursodeoxycholic acid | | UDCA | | 0.176 ±0.049 | 0.39 ±0.18 |
| α-Muricholic acid | | α-MCA | | 0.972 ±0.27 | 1.39 ±0.45 |
| β-Muricholic acid | | β-MCA | | 1.93 ±0.42 | 3.72 ±1.2 |
| ω-Muricholic acid | | ω-MCA | | 1.18 ±0.3 | 2.26 ±0.95 |
| **Bile Acid Glycine Conjugates** | |  | |  |  |
| Glycocholic acid | | GCA | | 0.746 ±0.38 | 0.405 ±0.1 |
| Glychochenodeoxycholic acid | | GCDCA | | 0.0184 ±0.0062 | 0.00929 ±0.0019 |
| Glycodeoxycholic acid | | GDCA | | 0.0226 ±0.0031 | 0.021 ±0.0059 |
| Glycohyodeoxycholic acid | | GHDCA | | 0.0122 ±0.0045 | 0.00869 ±0.0019 |
| Glycolithocholic acid | | GLCA | | 0.0255 ±0.0073 | 0.0125 ±0.0018 |
| Glycoursodeoxycholic acid | | GUDCA | | 0.0241 ±0.01 | 0.0144 ±0.0031 |
| **Bile Acid Taurine Conjugates** | |  | |  | |
| Taurocholic acid | | TCA | | 269 ±89 | 214 ±46 |
| Taurochenodeoxycholic acid | | TCDCA | | 17.9 ±6.6 | 8.38 ±1.2 |
| Taurodeoxycholic acid | | TDCA | | 22.8 ±3.6 | 19.6 ±6 |
| Taurolithocholic acid | | TLCA | | 2.3 ±0.44 | 1.47 ±0.39 |
| Tauroursodeoxycholic acid | | TUDCA | | 21.2 ±7 | 16.3 ±3.7 |
| Tauro-α-Muricholic acid | | T-α-MCA | | 27.7 ±9.6 | 17.4 ±2.4 |
| Tauro-β-Muricholic acid | | T-β-MCA | | 81.7 ±29 | 71.6 ±13 |
| Tauro-ω-Muricholic acid | | T-ω-MCA | | 71.5 ±22 | 55.8 ±11 |
| **Pseudo-quantiative Bile Acid Results (relative abundance)** | | | | | |
| **C27 Bile Acid Precoursors** | |  | |  |  |
| Dihydroxycholestanoic acid | | DiCSA(s) | | 59.9 ±13% | 36.3 ±13% |
| Trihydroxycholestanoic acid | | TriHCA(s) | | 18.1 ±13% | 7.47 ±1.7% |
| **Unconjugated Bile Acids** | |  | |  |  |
| Hyocholic acid | | HCA(s) | | 33.3 ±7.8% | 60.9 ±11% |
| Hyodeoxycholic acid | | HDCA(s) | | 13.3 ±3.6% | 36.5 ±16% |
| Murocholic acid | | MCA(s) | | 31.3 ±9.9% | 44.6 ±15% |
| **Conjugated Bile Acids** | |  | |  |  |
| Glycohyocholic acid | | GHCA(s) | | 23.8 ±12% | 12.6 ±4.5% |
| Taurohyocholic acid | | THCA(s) | | 25 ±12% | 12.1 ±4.2% |
|  |  | |  |  |  |
