# Supplementary material for: Perinatal Exposure of Mice to the Pesticide DDT Impairs Energy Expenditure and Metabolism in Adult Female Offspring
Source: PLoS One. 2014 Jul 30;9(7):e103337. doi: 10.1371/journal.pone.0103337 (PMC4116186; doi:10.1371/journal.pone.0103337)
Supplement: Supplementary file 6 [file pone.0103337.s003.docx]

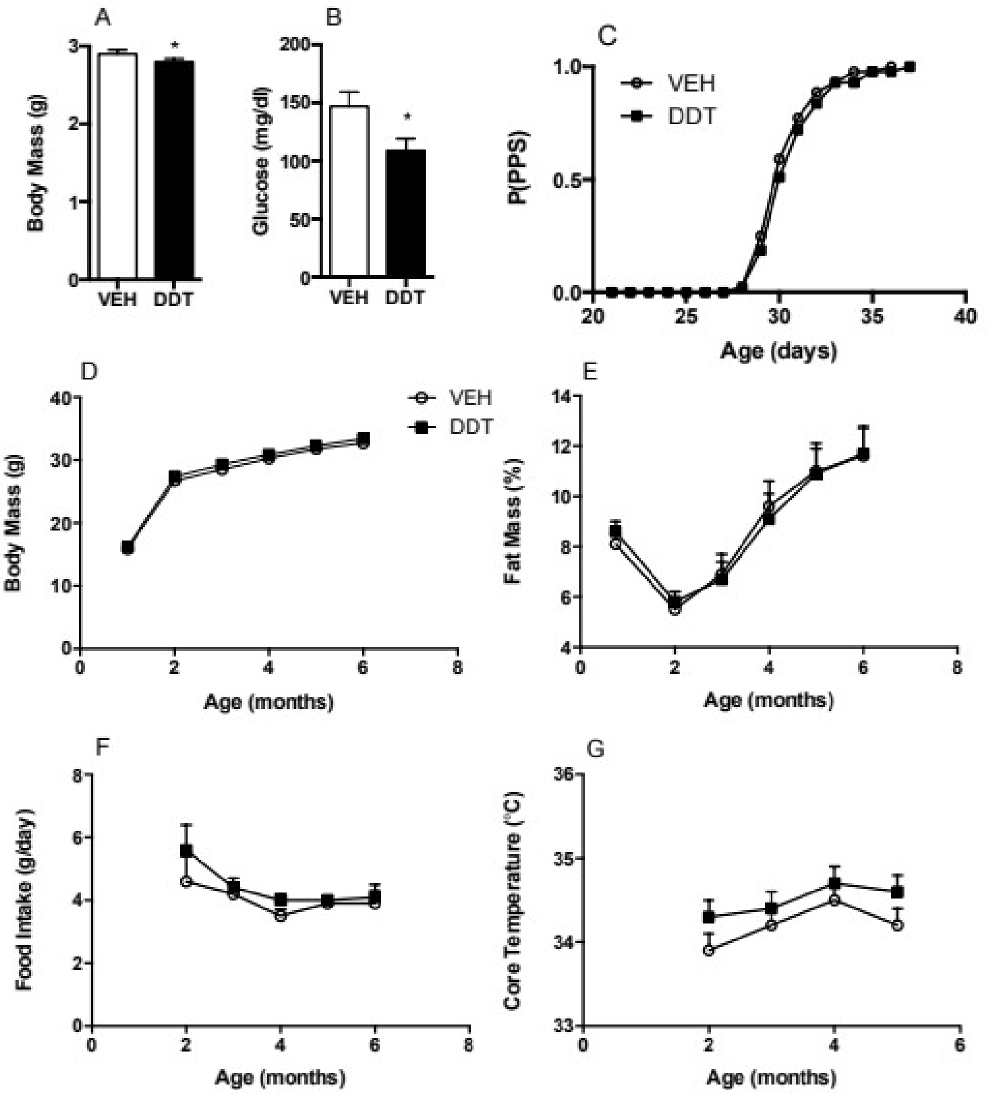

A
Body Mass (g)
VEH
DDT
B
Glucose (mg/dl)
VEH
DDT
C
P(PPS)
VEH
DDT
Age (days)
D
Body Mass (g)
VEH
DDT
Age (months)
E
Fat Mass (%)
Age (months)
F
Food Intake (g/day)
Age (months)
G
Core Temperature (°C)
Age (months)
