# Supplementary material for: Perinatal Exposure of Mice to the Pesticide DDT Impairs Energy Expenditure and Metabolism in Adult Female Offspring
Source: PLoS One. 2014 Jul 30;9(7):e103337. doi: 10.1371/journal.pone.0103337 (PMC4116186; doi:10.1371/journal.pone.0103337)
Supplement: Supplementary file 7 [file pone.0103337.s008.docx]

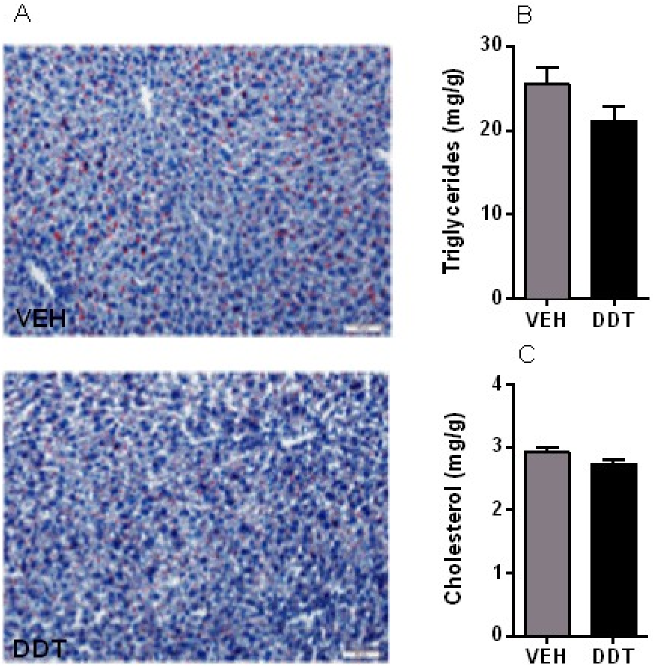
A
VEH
DDT
B
Triglycerides (mg/g)
30
20
10
0
VEH
DDT
C
Cholesterol (mg/g)
4
3
2
1
0
VEH
DDT
